# Zinc-Alpha 2-Glycoprotein Gene Expression in Adipose Tissue Is Related with Insulin Resistance and Lipolytic Genes in Morbidly Obese Patients

**DOI:** 10.1371/journal.pone.0033264

**Published:** 2012-03-19

**Authors:** Lourdes Garrido-Sánchez, Eduardo García-Fuentes, Diego Fernández-García, Xavier Escoté, Juan Alcaide, Pablo Perez-Martinez, Joan Vendrell, Francisco J. Tinahones

**Affiliations:** 1 CIBERDEM, Hospital Universitari Joan XXIII, Pere Virgili Institute, Tarragona, Spain; 2 Fundación IMABIS, Malaga, Spain; 3 Ciber Fisiopatología Obesidad y Nutrición (CIBEROBN), Malaga, Spain; 4 Servicio de Endocrinología y Nutrición, Hospital Clínico Virgen de la Victoria, Malaga, Spain; 5 Servicio de Medicina Interna, Hospital Universitario Reina Sofia, Córdoba, Spain; Postgraduate Medical Institute & Hull York Medical School, University of Hull, United Kingdom

## Abstract

**Objective:**

Zinc-α_2_ glycoprotein (ZAG) stimulates lipid loss by adipocytes and may be involved in the regulation of adipose tissue metabolism. However, to date no studies have been made in the most extreme of obesity. The aims of this study are to analyze ZAG expression levels in adipose tissue from morbidly obese patients, and their relationship with lipogenic and lipolytic genes and with insulin resistance (IR).

**Methods:**

mRNA expression levels of *PPARγ*, *IRS-1*, *IRS-2*, lipogenic and lipolytic genes and ZAG were quantified in visceral (VAT) and subcutaneous adipose tissue (SAT) of 25 nondiabetic morbidly obese patients, 11 with low IR and 14 with high IR. Plasma ZAG was also analyzed.

**Results:**

The morbidly obese patients with low IR had a higher VAT *ZAG* expression as compared with the patients with high IR (p = 0.023). In the patients with low IR, the VAT *ZAG* expression was greater than that in SAT (p = 0.009). ZAG expression correlated between SAT and VAT (r = 0.709, p<0.001). VAT *ZAG* expression was mainly predicted by insulin, HOMA-IR, plasma adiponectin and expression of adiponectin and *ACSS2*. SAT *ZAG* expression was only predicted by expression of *ATGL*.

**Conclusions:**

*ZAG* could be involved in modulating lipid metabolism in adipose tissue and is associated with insulin resistance. These findings suggest that *ZAG* may be a useful target in obesity and related disorders, such as diabetes.

## Introduction

Classically, obesity has been considered to be associated with a proinflammatory state, generating an increased incidence of dyslipidemia and insulin resistance (IR) [1]. This association seems to be mediated by the release of various proinflammatory adipokines and cytokines (e.g., leptin, adiponectin, TNF alpha, IL-6) by adipose tissue [2]–[4]. These proteins act either in an autocrine/paracrine/endocrine manner to locally regulate the adipocyte metabolism or as endocrine signals.

Zinc-α_2_ glycoprotein (ZAG), a protein secreted by different organs, such as liver, breast, lung and prostate [5], has recently been found to be expressed in both mature adipocytes [6], [7] and in visceral and subcutaneous adipose tissue in animal models (i.e., mice, rats) and humans [8], [9]. ZAG was initially identified as the lipid mobilizing factor associated with loss of adipose tissue in patients with cancer cachexia [10], [11]. Various studies have shown that ZAG gene expression is reduced in subcutaneous adipose tissue from obese persons [7], [12]. Moreover, studies in ZAG-deficient mice show that the intake of a standard or a high-fat diet (HFD) increases the body weight in comparison to identically treated wild-type mice [13]. Likewise, ZAG overexpression in HFD-fed obese mice results in a reduction of body weight, epididymal fat mass and percentage of epididymal fat [14].

Both *in vivo* and *in vitro* studies have shown that ZAG stimulates lipid loss by adipocytes [15], [16]. In addition, ZAG may also stimulate lipolysis through interaction with β3-adrenoreceptors, suggesting a role in lipid catabolism [10], [17]. The incubation of ZAG with adipocytes isolated from murine adipose tissue greatly stimulates glycerol release in a dose-dependent manner [10]. The lipolytic effect of ZAG on adipocytes [15], together with a high expression in adipose tissue during fat loss in patients with cancer cachexia [9], suggest that ZAG may be involved in the local regulation of adipose tissue metabolism.

Lipolysis in healthy subjects is extremely sensitive to the action of insulin. Recently, we have shown that the expression of lipogenic and lipolytic genes was altered in morbidly obese patients and was influenced by the adipose tissue location and clinical phenotype, such as IR [18]. Several studies have shown that ZAG gene expression is associated negatively with plasma insulin levels [7], [14], [19]. Mracek et al. [7] suggested that ZAG, as a major adipokine, may have a protective role in the susceptibility to obesity and its related IR. Furthermore, the overexpression of ZAG in 3T3-L1 adipocytes provokes an increased expression of adiponectin, which is highly involved in the control of IR [20]. This all suggests that ZAG may be a gene involved in the regulation of body weight and insulin sensitivity [15], [21].

Despite evidence from animal and human *in vivo* studies, few data exist about ZAG levels in the most extreme form of obesity. All the above evidence led us to hypothesize that ZAG gene expression in adipose tissue from morbidly obese patients may have a relationship with the expression of these lipogenic and lipolytic genes. To test this hypothesis, we analyzed ZAG gene expression levels in visceral and subcutaneous adipose tissue from a group of morbidly obese patients, and their relationship with lipogenic and lipolytic genes and with IR.

## Results

### Anthropometric and biochemical characteristics

Table 1 summarizes the characteristics of the morbidly obese patients according to whether they had low IR or high IR. Morbidly obese patients with high IR had a larger waist and higher serum levels of glucose, triglycerides, insulin, CRP and HOMA-IR (Table 1). Plasma adiponectin levels were significantly lower in the morbidly obese patients with high IR. Serum ZAG was similar between the two groups of morbidly obese patients.

**Table 1:** Anthropometric and biochemical variables in the morbidly obese patients classified according to their insulin resistance (IR).

	Low IR	High IR	P
**N (men/women)**	11 (5/6)	14 (7/7)	Ns
**Age (years)**	40.9±10.6	38.3±7.9	Ns
**Weight (kg)**	134.55±24.15	159.37±30.39	0.051
**BMI (Kg/m^2^)**	50.6±8.09	57.32±5.95	Ns
**Waist (cm)**	131.1±13.68	148.5±16.80	0.008
**Hip (cm)**	149.5±13.41	159.1±16.97	Ns
**Glucose (mmol/L)**	4.9±0.372	5.73±0.970	0.001
**Cholesterol (mmol/L)**	4.86±0.963	4.82±0.933	Ns
**Triglycerides (mmol/L)**	1.12±0.684	1.78±0.646	0.003
**FFA (mmol/L)**	0.437±0.147	0.540±0.180	Ns
**Insulin (pmol/L)**	91.04±24.93	293.14±95.35	<0.001
**HOMA-IR**	2.85±0.734	11.46±4.73	<0.001
**Leptin (ng/mL)**	61.82±31.89	64.82±25.34	Ns
**Adiponectin (ng/mL)**	10.21±2.95	7.03±4.40	0.025
**CRP (mg/L)**	3.88±3.25	10.15±9.14	0.042
**Serum ZAG (mg/L)**	36.21±10.33	37.62±9.64	Ns

The results are given as the mean ± SD. BMI: body mass index; FFA: Free fatty acids; HOMA-IR: homeostasis model assessment of insulin resistance index, CRP: C-reactive protein; ZAG: zinc alpha-2 glycoprotein. Ns: Not significant.

### ZAG gene expression according to insulin resistance

**Figure 1:**
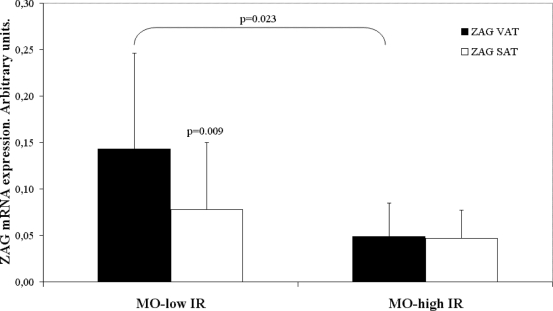
*ZAG* gene expression levels in visceral (VAT) (▪) and subcutaneous (SAT) (□) adipose tissue. MO low IR: morbidly obese persons with low insulin resistance. MO high IR: morbidly obese persons with high insulin resistance. Adipose tissue expression levels for each gene were normalized using cyclophilin A. The results are given as the mean ± SD.

The morbidly obese patients with low IR had a significantly higher *ZAG* gene expression in VAT as compared with the patients with high IR (p = 0.023) (Figure 1). In SAT, patients with low IR had higher *ZAG* gene expression though the difference was not significant (p = 0.232). No gender dimorphism was detected for ZAG gene expression.

In the morbidly obese patients with low IR, the ZAG gene expression in VAT was significantly greater than that in SAT (p = 0.009) (Figure 1). No significant differences were detected between VAT and SAT in the morbidly obese patients with high IR (p = 0.534) (Figure 1).

### Relation between ZAG gene expression and anthropometric and biochemical characteristics

**Figure 2:**
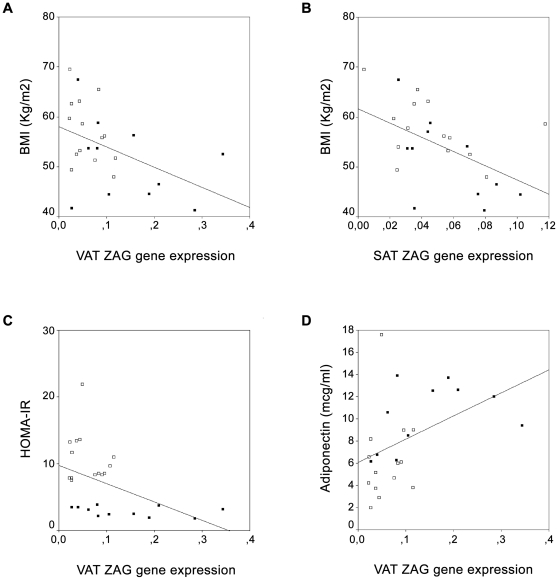
Correlations between *ZAG* gene expression levels in adipose tissue and anthropometric and biochemical variables. (A) Comparison of ZAG gene expression levels in visceral adipose tissue (VAT) and BMI. (B) Comparison of ZAG gene expression levels in subcutaneous adipose tissue (SAT) and BMI. (C) Comparison of ZAG gene expression levels in VAT and HOMA-IR. (D) Comparison of ZAG gene expression levels in VAT and adiponectin circulating levels. (▪) Morbidly obese persons with low insulin resistance. (□) Morbidly obese persons with high insulin resistance.

The serum ZAG levels did not correlate significantly with any of the anthropometric or biochemical variables studied (data not shown). *ZAG* gene expression levels in VAT and SAT correlated positively with age (r = 0.638, p = 0.001; and r = 0.580, p = 0.004, respectively). However, *ZAG* gene expression in VAT and SAT correlated negatively with weight (r = −0.585, p = 0.002; and r = −0.610, p = 0.002, respectively), BMI (r = −0.442, p = 0.036; and r = −0.524, p = 0.010 respectively) (Figure 2a, figure 2b) and waist circumference (r = −0.773, p<0.001; and r = −0.636, p = 0.001, respectively). *ZAG* gene expression in VAT correlated negatively with insulin (r = −0.565, p = 0.005) and HOMA-IR (r = −0.457, p = 0.029) (Figure 2c), and positively with the circulating adiponectin levels (r = 0.509, p = 0.013) (Figure 2e). The *ZAG* gene expression in VAT and SAT did not correlate significantly with any of the other anthropometric and biochemical variables studied (data not shown).

### Relationship between ZAG gene expression and lipid metabolism

The serum ZAG levels did not correlate significantly with the expression of any of the genes studied or the expression of ZAG in VAT and SAT (data not shown).

Associations between *ZAG* gene expression with other genes in VAT and SAT were explored. Z*AG* gene expression in VAT correlated strongly with *ZAG* gene expression in SAT (r = 0.709, p<0.001). In VAT, *ZAG* gene expression correlated significantly and positively with the expression of *PPARγ*, *ACSS2*, *DGAT1*, *ATGL*, *IRS-1*, *IRS-2* and adiponectin (Table 2). In SAT, *ZAG* gene expression correlated significantly and positively with *PPARγ*, *ACC1*, *DGAT1*, *ATGL*, *HSL* and adiponectin (Table 3).

**Table 2:** Bivariate correlations (*p*) between ZAG gene expression and other genes expression in visceral adipose tissue.

	ZAG
**PPARγ**	0.718 (0.001)
**ACC1**	Ns
**ACSS2**	0.689 (0.002)
**DGAT1**	0.689 (0.002)
**ATGL**	0.645 (0.005)
**HSL**	Ns
**Adiponectin**	0.684 (0.002)
**IRS-1**	0.616 (0.048)
**IRS-2**	0.886 (0.003)

R: Spearman's Rho; ACC1: acetyl-coenzime carboxilase 1; ACSS2: acyl-CoA synthetase short-chain family member 2; DGAT1: acyl Coenzyme A:cholesterol acyltransferase; ATGL: adipose triglyceride lipase; HSL: Hormose-sensitive lipase; IRS_1: insulin receptor substrate 1 IRS_2: insulin receptor substrate 2; PPARγ: peroxisome proliferator-activated receptor-γ, VAT: visceral adipose tissue; Ns: not significant.

**Table 3:** Bivariate correlations (*p*) between ZAG gene expression and other genes expression in subcutaneous adipose tissue.

	ZAG
**PPARγ**	0.541 (0.030)
**ACC1**	0.721 (0.002)
**ACSS2**	Ns
**DGAT1**	0.474 (0.048)
**ATGL**	0.638 (0.008)
**HSL**	0.568 (0.022)
**Adiponectin**	0.750 (0.001)
**IRS-1**	Ns
**IRS-2**	Ns

R: Spearman's Rho; PPARγ: peroxisome proliferator-activated receptor-γ, ACC1: acetyl-coenzime carboxilase 1; ACSS2: acyl-CoA synthetase short-chain family member 2; DGAT1: acyl Coenzyme A:cholesterol acyltransferase; ATGL: adipose triglyceride lipase; HSL: Hormose-sensitive lipase; IRS_1: insulin receptor substrate 1 IRS_2: insulin receptor substrate 2; SAT: subcutaneous adipose tissue; Ns: not significant.

In order to strengthen the independence of these associations as predictors of ZAG gene expression, a multiple regression analysis model was constructed for each depot. In the VAT depot model, age, BMI, waist circumference, insulin, circulating adiponectin levels, HOMA-IR and mRNA expression of genes for adiponectin, *ACSS2*, *DGAT1* and *ATGL* were selected as independent variables. VAT *ZAG* gene expression was mainly predicted (R^2^ = 0.959) by insulin (B = −0.088, p = 0.030), HOMA-IR (B = 0.320, p = 0.025), circulating adiponectin levels (B = 0.146, p = 0.045) and expression of adiponectin (B = 0.515, p = 0.039) and *ACSS2* (B = −1.346, p = 0.014). In the SAT model, age, BMI, waist circumference, HOMA-IR and mRNA expression of adiponectin, *ACC1*, *DGAT1*, *ATGL* and *HSL* were included as independent variables. SAT *ZAG* gene expression (R^2^ = 0.980) was only predicted by *ATGL* gene expression (B = 0.272, p = 0.021).

## Discussion

This study is the first to analyze the ZAG expression in the most extreme form of obesity. We show that VAT and SAT ZAG expression and its relation with the insulin resistance is different. We found a inverse relation between the degree of IR and the ZAG gene expression in VAT. Furthermore, the ZAG gene expression showed a direct relation with the genetic expression of lipolytic enzymes in both VAT and SAT.

No significant differences were found between morbidly obese patients with low IR and those with high IR in the circulating serum ZAG levels. These findings corroborate those of Stejskal et al. [22], who found no differences in serum ZAG concentrations between obese patients with the metabolic syndrome and otherwise healthy controls. Another study also failed to find significant differences in serum ZAG concentrations between overweight patients and obese patients, nor between patients with and without the metabolic syndrome [23]. However, recent studies in humans have reported reductions in serum ZAG levels in obese patients [14], [19]. Given that ZAG is produced by several different tissues [8], serum levels may be influenced by the secretion of each particular tissue. In addition, the clearance of ZAG in the circulation might be altered in obesity.

In our study ZAG gene expression in VAT and SAT was inversely associated with different anthropometric variables, such as weight, BMI and waist circumference. Previous studies have shown a down-regulation of the expression of this adipokine in obese patients [12], [24]. We show that even in the morbidly obese patients, the inverse relationship between BMI and ZAG gene expression is maintained. Studies in mice show that the overexpression of ZAG is associated with a reduced body weight and percentage of epididymal fat [14]. Animal studies have demonstrated a beneficial effect of ZAG protein administration on reducing body weight by decreasing fat content in mice, even though they maintained normal eating habits [10]. Although ZAG gene expression has been shown to be inversely related to adiposity, its regulation in obesity remains to be established.

In this study we noted a reduction in ZAG gene expression in the morbidly obese patients with high IR, though the difference was not significant in VAT. The negative association found between IR and ZAG gene expression in VAT is in agreement with other studies [7], [14], [23]. Mracek et al. [7] suggested that ZAG may have a protective role in the susceptibility to obesity and its related IR. Others have demonstrated that treatment with ZAG stimulates the use of glucose and increases lipid oxidation in different murine tissues [17]. The positive association found between the expression of ZAG gene and IRS-1 and IRS-2 strengthens the case for the implication of ZAG in mechanisms regulating IR. It is well accepted that IRS-1 is a main component in the activation of insulin signalling in adipose tissue [25]. Different studies have shown a lower amount of IRS-1 in 30% of subjects at high risk for type 2 diabetes, such as first-degree relatives of type 2 diabetic and obese subjects [26], [27]. In earlier studies we found elevated levels of IRS-1 gene expression in morbidly obese patients with low IR compared with those with high IR [28].

In this context, our results show that IR and plasma adiponectin circulating levels and expression are the main variables significantly associated with the ZAG gene expression levels. As in other studies made in non-morbidly obese patients [7], [23], our results show a positive association between ZAG gene expression, in both VAT and SAT, and the adiponectin gene expression levels in morbidly obese patients. In animal models of type 2 diabetes a role for ZAG as a modulator of adipocyte endocrine signalling at a local site has been proposed. In 3T3-L1, the overexpression of ZAG leads to an increased expression of adiponectin [21]. In addition, a positive association has also been found between ZAG and PPARγ gene expression. This nuclear receptor is involved in the regulation of ZAG synthesis [6]. It has previously been shown that treatment with rosiglitazone, a selective PPARγ agonist, induced an increase in ZAG and adiponectin gene expression levels in human adipocytes [6]. These findings all suggest the existence of a regulatory mechanism between both adipokines acting in a coordinated manner, with potential implications in obesity and type 2 diabetes mellitus [29].

The underlying mechanisms for the different findings in VAT compared to SAT are unknown. ZAG gene expression was greater in VAT than in SAT, but only in morbidly obese patients with a low IR. The endocrine function, and response to insulin and other hormones differ between SAT and VAT. These actions are also markedly altered in the adipose tissue of obese persons [30]. This might explain the tissue-specific expression of ZAG gene. This different expression may also be related with the concept that VAT contributes to the morbidity associated with the metabolic syndrome features.

Several key enzymes in lipid metabolism were determined in VAT and SAT of morbidly obese patients. Our results also suggest that ZAG may be involved in the regulation of lipid metabolism. ZAG gene expression in morbidly obese patients is positively associated with the expression of lipogenic (ACC1, ACSS2 and DGAT1) and lipolytic (ATGL and HSL) genes in VAT and SAT. We have previously shown in slim and overweight persons an association of ZAG gene expression with the expression of lipolytic genes [23]. Studies in ZAG overexpression mice show a decrease in FAS, ACC1 and DGAT mRNA and a increase in HSL mRNA in epididymal adipose tissue [14]. The discrepancy found in the relation between ZAG gene expression and the lipogenic enzymes may be due to the type of sample studied. Our study was undertaken in persons with extreme obesity, in which we had earlier shown that the expression of both lipogenic and lipolytic genes was significantly raised [18].

Different studies have shown that treatment with ZAG stimulates lipolysis *in vitro* in a dose-dependent manner [10], [31]. The mobilization of stored triglycerides from adipose tissue is mediated mainly by the activation of two genes: HSL and ATGL [30]. HSL was elevated in adipose tissue from ZAG-overexpressing transgenic mice which exhibit decreased body weight and epididymal fat [14]. *In vivo*, the administration of ZAG to mice induces a reduction in body fat and an increase in serum FFA by HSL activation [15], [16]. Although HSL was initially considered to be the rate-limiting enzyme for lipolysis, recent data suggest that ATGL may also be rate limiting [32]. In this context, SAT from morbidly obese patients showed that, although ZAG and HSL gene expression are positively correlated, ATGL gene expression is the most important determinant of ZAG gene expression in these patients. Our results suggest that ZAG gene expression might be related to its function in modulating lipid metabolism [33].

In conclusion, this study adds to previous results obtained in non-morbidly obese patients. ZAG is closely linked to obesity. The ZAG gene expression may be involved in the regulation of lipid metabolism in morbidly obese patients, but all of them (ZAG+lipogenic and lipolytic genes) also could be regulated through the same signalling pathway, meaning that ZAG does not regulates these genes. The relationship between ZAG gene expression and IR and adiponectin in human adipose tissue reinforces previous experimental data and warrants further mechanistic studies as a useful target in obesity and related disorders, such as diabetes.

## Materials and Methods

### Subjects

The study included 25 nondiabetic morbidly obese patients (body mass index (BMI) 57.4±5.2 Kg/m^2^), 11 with low IR (homeostasis model assessment of insulin resistance index (HOMA-IR)<4.7) and 14 with high IR (HOMA-IR>8) [18], [29]. The cut-off points for the HOMA-IR were taken from previous studies carried out in a healthy population with no carbohydrate metabolism disorders [18], [29]. All the patients underwent biliopancreatic diversion of Scopinaro. Patients were excluded if they had type 2 diabetes mellitus, cardiovascular disease, arthritis, acute inflammatory disease, infectious disease, or were receiving drugs that could alter the lipid profile or the metabolic parameters at the time of inclusion in the study. The weight of all the persons had been stable for at least one month and none had renal involvement. All participants gave their informed consent and the study was reviewed and approved by the Ethics and Research Committee of Virgen de la Victoria Clinical University Hospital, Malaga, Spain.

### Laboratory measurements

Blood samples were collected after a 12-hour fast. The serum was separated and immediately frozen at −80°C. Serum biochemical parameters were measured in duplicate. Serum glucose, cholesterol, HDL cholesterol, triglycerides (Randox Laboratories Ltd., Antrium, UK) and free fatty acids (FFA) (WAKO Chemicals, Richmond, VA) were measured by standard enzymatic methods. Adiponectin levels were measured by enzyme immunoassay (ELISA) kits (DRG Diagnostics, Marburg, Germany). High-sensitivity C-reactive protein (CRP) levels were measured by ELISA kit from BLK Diagnostics (Badalona, Spain). Leptin levels were measured by ELISA kit from Mediagnost (Reutlingen, Germany). The insulin was analyzed by an immunoradiometric assay (IRMA) (BioSource International, Camarillo, CA), showing a 0.3% cross-reaction with proinsulin. Plasma ZAG levels were measured by sandwich ELISA (Bio-Vendor Laboratory Medicine, Inc., Palackeho, Czech Republic). The HOMA-IR was calculated from fasting insulin and glucose with the following equation: HOMA-IR = fasting insulin (µIU/mL)×fasting glucose (mol/L)/22.5.

### Visceral and subcutaneous adipose tissue mRNA

We analyzed the relative basal mRNA expression levels of *ZAG* and lipogenic and lipolytic genes in epiploic visceral adipose tissue (VAT) and abdominal subcutaneous adipose tissue (SAT). VAT and SAT were obtained during bariatric surgery in the morbidly obese patients [18], [34]. The biopsy samples were washed in physiological saline and immediately frozen in liquid nitrogen. Biopsy samples were maintained at −80°C until analysis. Frozen adipose tissue was homogenized with an Ultra-Turrax 8 (Ika, Staufen, Germany). Total RNA was extracted by RNeasy lipid tissue midi kit (QIAGEN Science, Hilden, Germany), and total RNA was treated with 55 U RNase-free deoxyribonuclease (QIAGEN Science, Hilden, Germany) following the manufacturer's instructions. The purity of the RNA was determined by the absorbance260/absorbance280 ratio on the Nanodrop ND-1000 spectrophotometer (Thermo Fisher Scientific Inc. Waltham, MA). The integrity of total purified RNA was checked by denaturing agarose gel electrophoresis and ethidium bromide staining. Total RNA was reverse transcribed to cDNA by using a high-capacity cDNA reverse transcription kit with RNase inhibitor (Applied Biosystems, Foster City, CA). The cDNA was used for quantitative real-time PCR with duplicates. The amplifications were performed using a MicroAmp® Optical 96-well reaction plate (Applied Biosystems, Foster City, CA) on an ABI 7500 Fast Real-Time PCR System (Applied Biosystems, Foster City, CA). RT-qPCR reactions were carried out for all genes using specific TaqMan® Gene Expression Assays (Applied Biosystems, Foster City, CA). We analyzed the relative basal mRNA expression levels of *ZAG* (Hs00426651_m1, RefSeq. NM_00185.3), adiponectin (Hs00605917_m1, RefSeq. NM_001177800.1 and NM_004797.3), insulin receptor substrate 1 (*IRS-1*) (Hs00178563_m1, RefSeq. NM_005544.2), insulin receptor substrate 2 (*IRS-2*) (Hs00275843_s1, RefSeq. NM_003749.2), peroxisome proliferator-activated receptor-γ (*PPARγ*) (Hs00234592_m1, RefSeq. NM_005037.5, NM_015869.4, NM_138711.3 and NM_138712.3) and genes involved in lipogenesis or lipolysis: acyl-CoA:cholesterol acyltransferase (*DGAT1*) (Hs00201385_m1, refSeq. NM_012079.4), acetyl-CoA carboxilase 1 (*ACC1*) (Hs00167385_m1, RefSeq. NM_ 198834.1, NM_198836.1, NM_198837.1, NM_198838.1 and NM_198839.1), acyl-CoA synthetase short-chain family member 2 (*ACSS2*) (Hs00218766_m1, RefSeq. NM_001076552.2, NM_018677.3 and NR_028046.1), adipose triglyceride lipase (*ATGL*) (Hs00386101_m1, RefSeq. NM_020376.3) and hormone-sensitive lipase (*HSL*) (Hs00193510_m1, RefSeq. NM_005357.2). The cycle threshold (Ct) value for each sample was normalized with the expression of cyclophilin A (*PPIA*) (4326316E, RefSeq. NM_021130.3). During PCR, the Ct values for each amplified product were determined using a threshold value of 0.1. SDS software 2.3 and RQ Manager 1.2 (Applied Biosystems, Foster City, CA) were used to analyze the results with the comparative Ct method (2^−ΔΔCt^). All data were expressed as an n-fold difference relative to the calibrator (a mixture of the SAT and VAT tissues was used as the calibrator sample).

### Statistical analysis

Based on previous results from our group [18], the study was designed to make a comparative analysis between the two study groups, with a standard deviation of the PPARγ mRNA expression of 0.65, a capacity to detect a change in PPARγ mRNA expression of 0.90 and a detection power of 80%. For an α = 0.05, the minimum sample size needed for each group was 10 cases. The statistical analysis was done with SPSS (Version 11.5 for Windows; SPSS, Chicago, IL). Because most of the parameters analyzed do not have a normal distribution, we used non-parametric tests. Comparison between the results of the morbidly obese patients with low or high IR was made with the Mann-Whitney test. The Spearman correlation coefficients were calculated to estimate the correlations between variables. Multiple linear regressions were used to determine the association between variables. Values were considered to be statistically significant when the *p*≤0.05. The results are given as the mean ± SD.
